# Concealed use of herbal and dietary supplements among Thai patients with type 2 diabetes mellitus

**DOI:** 10.1186/s40200-017-0317-3

**Published:** 2017-08-23

**Authors:** Prapaipan Putthapiban, Weera Sukhumthammarat, Chutintorn Sriphrapradang

**Affiliations:** 1 Department of Medicine, Faculty of Medicine Ramathibodi Hospital, Mahidol University, 270 Rama VI Road Bangkok, Bangkok, 10400 Thailand; 2 Faculty of Science, Mahidol University, Bangkok, 10400 Thailand; 3 Faculty of Dentistry, Mahidol University, Bangkok, 10400 Thailand

**Keywords:** Asia, Complementary therapies, Diabetes mellitus, herb-drug interactions, Medicinal plants

## Abstract

**Background:**

Diabetes mellitus (DM) has been one of the most common chronic diseases that create great impacts on both morbidities and mortalities. Many patients who suffering from this disease seek for complementary and alternative medicine. The aim of this study was to determine the prevalence and related factors of herbal and dietary supplement (HDS) use in patients with DM type 2 at a single university hospital in Thailand.

**Methods:**

A cross-sectional study was performed in 200 type 2 DM patients via face-to-face structured interviews using developed questionnaires comprised of demographic data, diabetes-specific information, details on HDS use, and medical adherence.

**Results:**

From the endocrinology clinic, 61% of total patients reported HDS exposure and 28% were currently consuming. More than two-thirds of HDS users did not notify their physicians, mainly because of a lack of doctor concern; 73% of cases had no awareness of potential drug-herb interaction. The use of drumstick tree, turmeric and bitter gourd and holy mushroom were most frequently reported. The main reasons for HDS use were friend and relative suggestions and social media. Comparisons of demographic characteristics, medical adherence, and hemoglobin A1c among these non-HDS users, as well as current and former users, were not statistically significantly different.

**Conclusions:**

This study revealed a great number of DM patients interested in HDS use. The use of HDS for glycemic control is an emerging public health concern given the potential adverse effects, drug interactions and benefits associated with its use. Health care professionals should aware of HDS use and hence incorporate this aspect into the clinical practice.

**Electronic supplementary material:**

The online version of this article (doi:10.1186/s40200-017-0317-3) contains supplementary material, which is available to authorized users.

## Background

Complementary and alternative medicine (CAM) refers to various types of medical practices and products apart from currently considered conventional medicine [1]. The National Center for Complementary and Integrative Health, the U.S. government’s lead agency for scientific research on CAM, classifies CAM into three subgroups: natural products, mind and body practices, and other approaches such as Ayurvedic medicine and traditional Chinese medicine [1]. The overall use of CAM in the United States has been studied for over a decade by the National Health Interview Survey and has shown a dramatic increase with a maximum of 35% in 2007 [2, 3]. A prevalence of CAM use in diabetes has been analyzed in several studies, and the results were within a range of 34–57% in the United States [4–7]. The prevalence of CAM use was even as high as 76% in South Asians [8]. Adults who have chronic diseases including diabetes mellitus (DM) tend to use CAM more than healthy people [9–11]. In fact, a previous survey reported that adults with diabetes were 1.6 times more likely to use CAM than the non-diabetes population [12]. Biologically based practices such as consumption of herbal and dietary supplements (HDS) were the most widely used modalities of CAM [2, 6, 9, 12]. Previous studies revealed that female gender, higher educational status, duration of disease, and the elderly has a significant association with CAM use in both Western and Asian countries [7, 8, 13]. The major purposes of CAM use were to improve health and well-being or to alleviate undesired symptoms from chronic illness [14, 15]. Interestingly, recommendation by family or friend was the most common reason for CAM use in Thailand [16–18]. Many scientific studies have shown the effectiveness of herbal remedies in lowering plasma glucose both in vitro and in vivo [19–21]. A randomized, double-blinded, placebo-controlled trial indicated the efficacy of curcumin extract in preventing a progression from prediabetic state to type 2 DM [22]. Despite the growing use of HDS, a well-designed long-term clinical trial on safety and adverse effects has not been established yet [19]. Furthermore, the issue of drug interaction is also unclear [23].

The purpose of the study was to determine the prevalence and pattern of HDS use in type 2 diabetic patients. Demographic characteristics, reasons for HDS usage, frequency of revealed HDS use to physicians, and the relationship between glycemic status and conventional medical adherence with HDS use were extensively explored in this study.

## Methods

A cross-sectional survey was conducted from July through October 2015 using face-to-face structured interviews at the Endocrine Clinic in Ramathibodi Hospital, Bangkok, Thailand. The study was approved by the Ethics Committee of Ramathibodi Hospital.

The questionnaire design was developed following a comprehensive literature review of related studies. An initial pilot study was applied to 20 subjects to determine understanding of the questions and validity. The final version of the questionnaire comprised four domains: demographic data, diabetes-specific information, details on HDS use, and conventional anti-diabetic medication adherence (see Additinal file 1). Demographic characteristics included age, gender, hometown, education, employment status, marital status, religion, family type, personal income, exercise, smoking status and alcohol consumption. Diabetes-specific information included duration of DM, presence of self-monitoring of blood glucose (SMBG), diabetes- related complications and hospitalizations, other comorbidities, types of conventional anti-diabetic medication, last fasting plasma glucose (FPG) and hemoglobin A1c (HbA1c), and serum creatinine. HDS usage consisted of type of HDS use, medical purpose, dosage form, duration of HDS use, influencing factors and reasons for HDS use, access to HDS, expense of HDS in Thai bath per month, perception of both positive and negative effect of HDS, awareness of interaction between HDS and conventional medication and disclosure of HDS use to their physicians. The last section assessed the conventional anti-diabetic medication adherence by using the Thai version of the 8-item Morisky Medication Adherence Scale (MMAS-8) in patients with type 2 DM [24]. Respondents were considered as high, medium and low adherence according to an MMAS-8 score of 8, 6 to <8 and <6 points, respectively.

Inclusion criteria were adults aged above 18 years with a previous diagnosis of type 2 DM according to American Diabetes Association criteria [25]. Exclusion criteria were an inability to communicate or give consent, or not currently taking conventional anti-diabetic medication. All included patients were randomly approached to explain the purpose of the study, the procedure and their right to withdraw from the study at any time. The participants were given assurance of confidentiality of survey data. Informed consent was obtained from each of the eligible patients. Two authors of this study (P.P. and W.S.), both trained physicians, conducted the face-to-face structured interviews, with an average duration of 15–20 min. Medical records were reviewed in order to validate diabetes-specific information and laboratory investigation results.

Statistical analysis was performed using SPSS version 21 (IBM, Armonk NY, USA). Descriptive analysis was applied to assess prevalence and data related to HDS usage. Data were presented as mean ± SD or median (interquartile range, IQR), depending on the normality of data. *Chi*-square tests and ANOVA were used to chart comparisons of demographic data among three assigned subgroups according to HDS use (i.e. never, current and former use). A *p*-value of 0.05 or less determined statistical significance.

## Results

A total of 212 patients were invited to participate in the study. Five patients did not give their consent due to insufficient time, and 7 patients were excluded because of incomplete data. A total of 200 participants were included in the final analysis, 118 (59%) were females. The median age (IQR) was 65 (58–73) years. Among the participants, 69.5% lived in Bangkok and vicinity, 66.5% were retired, 71.5% were married and 93.5% were Buddhist.

Socio-demographic and disease-specific characteristics are displayed in Table 1, which classifies subjects into three groups according to their HDS use, i.e. non-HDS, current and former HDS users. Prevalence of HDS use in this study population was 61%; 56 patients (28%) were currently using HDS. There were no statistically significant differences among these three groups regarding sex, body mass index, education, occupation, monthly income, marital status, family type, smoking and alcohol drinking status. Furthermore, diabetes specific data - including duration of DM, diabetes-related complications, hospitalization due to diabetic emergency, conventional anti-diabetic treatment, SMBG, FPG, HbA1c, serum creatinine and medication adherence – revealed no statistically significant factors associated with HDS use.

**Table 1:** Comparison of characteristics among non-HDS users, current HDS users and former HDS users

Characteristics	Non HDS users (*n* = 78)	Current HDS users (*n* = 56)	Former HDS users (*n* = 66)	*p*-value
Age, yr. (IQR)^a^	66.0 (56.0–76.3)	64.0 (59.0–70.0)	65 (59.0–73.0)	0.660
Female (%)	42 (53.8%)	33 (58.9%)	43 (65.2%)	0.389
Overweight/obesity (%)	67 (85.9%)	47 (83.9%)	53 (80.3%)	0.663
Education (%)				0.747
Primary/secondary school Higher education	55 (70.5%) 23 (29.5%)	36 (64.3%) 20 (35.7%)	45 (68.2%) 21 (31.8%)	
Unemployed (%)	52 (66.7%)	36 (64.3%)	45 (68.2%)	0.901
Monthly income (%)				0.29
< 10,000 baht^b^ 10,000–30,000 baht > 30,000 baht	22 (28.2%) 40 (51.3%) 16 (20.5%)	23 (41.1%) 22 (39.3%) 11 (19.6%)	27 (40.9%) 23 (34.8%) 16 (24.2%)	
Marital status (%)				0.908
Married Not married	57 (73.1%) 21 (26.9%)	39 (69.6%) 17 (30.4%)	47 (71.2%) 19 (28.8%)	
Family type (%)				0.211
Alone Small, nuclear Large	9 (11.5%) 59 (75.6%) 10 (12.8%)	1 (1.8%) 46 (82.1%) 9 (16.1%)	3 (4.5%) 54 (81.8%) 9 (13.6%)	
Smoking status (%)				0.247
Never Former Current	60 (76.9%) 16 (20.5%) 2 (2.6%)	40 (71.4%) 10 (17.9%) 6 (10.7%)	50 (75.8%) 14 (21.2%) 2 (3.0%)	
Alcohol status (%)				0.386
Never Former Current	57 (73.1%) 13 (16.7%) 2 (2.6%)	36 (64.3%) 11 (19.0%) 9 (16.1%)	52 (78.8%) 4 (6.1%) 10 (15.2%)	
Duration of DM, yr. (SD)^a^	14.12 (10.39)	14.76 (9.18)	15.05 (9.05)	0.838
Diabetes complications (%)	55 (70.5%)	39 (69.6%)	43 (65.2%)	0.770
Hospitalization due to diabetic emergency (%)	12 (15.4%)	4 (7.14%)	12 (18.2%)	0.195
Treatment (%)				0.133
Oral meds only Insulin only Both	45 (57.7%) 13 (16.7%) 20 (25.6%)	34 (60.7%) 4 (7.1%) 18 (32.1%)	32 6 28	
SMBG (%)	43 (55.1%)	33 (58.9%)	39 (59.1%)	0.863
FPG, mg/dL (IQR)^a^	142 (112.8–180)	135.0 (111–166.3)	137 (110.3–172.5)	0.726
HbA1c, % (IQR)^a^	7.14 (6.5–8.4)	7.4 (6.9–8.4)	7.3 (6.7–8.3)	0.427
Serum creatinine, mg/dL (IQR)^a^	1.1 (0.8–1.5)	1.0 (0.8–1.4)	1.0 (0.7–1.5)	0.659
Low medical adherence^c^ (%)	20 (25.6%)	15 (26.8%)	18 (27.3%)	0.995

*Abbreviations: DM* diabetes mellitus, *FPG* fasting plasma glucose, *HbA1c* hemoglobin A1c, *HDS* herbal and dietary supplements, *IQR* interquartile range, *SD* standard deviation, *SMBG* self-monitoring of blood glucose

^b^1 USD is approximately 35.5 Thai baht

^c^ Thai version of 8-item Morisky Medication Adherence Scale was used to determine medication adherence. Low, medium and high adherence was defined as MMAS <6, 6 to <8, 8, respectively

^a^Data presented as mean ± SD

Of the 122 patients exposed to HDS, 84 (69%) reported using HDS continuously. The median (IQR) duration of HDS use was 6 (2–12) months. Processed herbs in capsule, tablet or potion form (41.9%) was the most common mode of HDS use. Other forms of HDS use were dried herbs and tea (34.3%) and raw unprocessed herbs (23.8%). As many as 248 types of HDS were found among the 122 patients who reported HDS use. *Moringa oleifera Lam.* (drumstick tree, 6.9%), *Curcuma longa L.* (turmeric, 4.9%), *Momordica charantia L.* (bitter gourd, 4.0%) and *Ganoderma lucidum* (holy mushroom, 3.6%) were the most commonly used herbs. Table 2 describes the frequency of HDS use among these patients. Surprisingly, nearly 8% of HDS were unknown, as patients were not aware of the ingredients in their HDS products.

**Table 2:** Frequency of herbal and dietary supplement (HDS) use

Herb	Frequency (%)
*Moringa oleifera* Lam.	17 (6.9)
*Curcuma longa* L.	12 (4.9)
*Momordica charantia* L.	10 (4.0)
*Ganoderma lucidum*	9 (3.6)
*Andrographis paniculata*	7 (2.8)
*Thunbergia laurifolia* Lindl.	6 (2.4)
*Annona muricata* L.	6 (2.4)
*Tinospora crispa*	4 (1.6)
*Gynostemma pentaphyllum*	4 (1.6)
*Helicteres isora* L.	4 (1.6)
*Cinnamomum* spp.	4 (1.6)
*Tradescantia fluminensis*	4 (1.6)
*Stevia rebaudiana* Bertoni	4 (1.6)
Unknown type	19 (7.7)
Others	138 (55.7)
Total^a^	248 (100)

^a^A patient may use more than one type of HDS, so these total more than 122

Reasons for HDS use and sources of HDS are shown in Table 3. Friend/family recommendation was the greatest motivation for HDS use, which accounted for nearly 50%. In addition, 13.9% reported that their friends and family also provided HDS for them. Information from the internet and media such as television, radio and social network (21.1%) was the second most influential factor regarding HDS use. Other motives were treatment of a chronic condition (11.2%), perception that HDS were safer than conventional medications (5.6%), disappointment from conventional medicine (4.4%), and promotion of general health and well-being (4.4%). Only 1.2% reported that a health care provider’s recommendation was their reason for HDS use. The majority of patients obtained HDS from a drug store and/or folk remedy shop (36.1%). Markets (17.2%), direct selling (16.4%), and collecting from their own garden were other source of HDS.

**Table 3:** Reasons for HDS use and sources of HDS

	Frequency (%)
Reason (*n* = 161)^a^	
Friend/family recommendation	79 (49.1)
Information from media and internet	34 (21.1)
Treatment of chronic condition	18 (11.2)
Safer than conventional practitioners	9 (5.6)
Disappointment from conventional medicine	7 (4.3)
General health and well-being	7 (4.3)
Treatment of acute condition	5 (3.1)
Health care provider’s recommendation	2 (1.2)
Source of HDS (*n* = 122)	
Drug store or folk remedy shop	44 (36.1)
Market	21 (17.2)
Direct selling	20 (16.4)
Collecting from their own garden	17 (13.9)
Provided by friends or family	17 (13.9)
Hospital	3 (2.5)

^a^More than one answer was applicable, so these total more than 122

If patients take a combination of conventional medication and HDS, awareness of drug-herb interaction and revealing their use to a physician are crucial. Nevertheless, only 7 patients (5.7%) reported an awareness of drug-herb interaction (Table 4). The majority of patients (73%), including both current and former HDS users were unaware of drug-herb interaction. Moreover, 85 out of 122 patients exposed to HDS (69.7%) did not inform their physicians about their use of HDS. The most common reason for their undisclosed use of HDS was that their doctor did not ask (47.1%). Additionally, 32.9% believed that there was no need to inform their physician, and 20% were afraid that their doctor would be against their HDS use.

**Table 4:** Awareness of interaction between conventional medicine and herbs, disclosure of HDS use to their physicians, effects reported by patients, and expense of HDS

	Total (%) *n* = 122	Current use *n* = 56	Former use *n* = 66	*p* - value
Awareness of interaction				0.036*
Yes Probably No	7 (5.7) 26 (21.3) 89 (73.0)	0 14 42	7 12 47	
Informed their physician about HDS use				0.844
Yes No	37 (30.3) 85 (69.7)	16 40	21 45	
Reasons for undisclosed use of HDS				0.469
Their physician doesn’t ask No need to inform their physician Their physician will disapprove of HDS use	40 (32.8) 28 (23.0) 17 (13.9)	19 12 9	21 16 8	
Effects reported by patients				
No change	47 (38.5)	17	30	0.037*
Positive effects^a^	66 (54.1)	37	29	
Negative effects^b^	9 (7.4)	2	7	
Expenditure on HDS, baht (IQR)^c^	300 (100–700)	295 (100–500)	300 (7.5–1037.5)	0.920

*Abbreviations: HDS* herbal and dietary supplements, *IQR* interquartile range

* Statistically significant at *p*-value <0.05

^a^Positive effects include strengthening of the body, good psychological condition and relief of several symptoms

^b^Negative effects include worsening of physical or psychological condition

^c^1 USD = approximately 35.5 Thai baht

Sixty-six patients out of a total of 122 patients (54.1%) who were exposed to HDS experienced positive effects from their HDS use. These positive effects were strengthening of the body, improved psychological condition, or relief of severe symptoms. A group of 47 patients (38.5%) reported no change in their health, however, 17 of these patients were continuing to use HDS. In addition, 9 patients (7.4%) suffered from negative effects, including worsening of physical psychological condition. The expense of HDS varied extensively, from obtaining HDS for no cost to spending up to 60,000 Thai baht (approximately 1690 USD; 1 USD = 35.5 Baht). The median (IQR) expenditure for HDS was 300 (100–700) baht.

A comparison between current and former HDS users found that former users were significantly aware of drug- herb interaction compared with current users (*X* ^2^ = 6.66, *p* = 0.036). Additionally, self-reported HDS effects (i.e. no change, positive and negative effects) were significantly difference within those two groups (*X* ^2^ = 10.25, *p* = 0.037). Other comparisons revealed no statistically significant differences.

## Discussion

The study showed that 61% of participating DM patients reported exposure to HDS since the time of first diagnosis with DM. This finding was higher than in a previous survey in Thai patients with chronic kidney disease, which found a prevalence of HDS use of 45% [17]. The prevalence of HDS use in other Asian countries, e.g. Sri Lanka (76%), India (67.7%) and Singapore (76%) was estimated to be greater than in the present survey [8, 26, 27]. In contrast, there was less frequent HDS use found in European countries as well as the United States [4–7, 28, 29]. For example, a survey in Norway reported only 33% HDS use in DM patients [28]. The reason for more commonly use HDS in Asian countries could be due to cultural perception of HDS.

These study findings revealed extremely varied types of HDS use. The most frequently used are *Moringa oleifera* Lam (6.9%), *Curcuma longa* L. (4.9%) and *Momordica charantia* L. (4%). *Moringa oleifera* was proved anti-hyperglycemic properties by lowering FPG and HbA1c in both animal studies [30, 31] and human studies [32, 33]. *Curcuma longa* L*.*, generally known as turmeric, is also an abundantly used HDS. Several studies reported anti-inflammatory and anti-hyperglycemic effects of *Curcuma longa L.* by down-regulating inflammatory cytokines [34–36]. Furthermore, it also prevents development of DM in prediabetic individuals [22]. Nevertheless, there have been only a limited numbers of studies on the adverse effects and toxicities of these herbs, thus demanding an urgent need to explore the safety issues [37].

From our study, processed herbs in capsule, tablet or potion form was the most frequent mode of HDS use (41.9%). Qualified preparation processes of these products are uncertain. Therefore, contamination and adulteration are of immense concern, as many studies have reported [38–40]. Dust, pollen, microbes, pesticides, toxic heavy metals and even prescription drugs were found in herbal medicinal products and caused adverse reactions [40]. In addition, of the 248 types of HDS, 19 (7.7%) were noted as unknown kinds of herbs, which leads to difficulty in monitoring toxicity and interaction between herbs and conventional medicine.

Recommendation by friends and family had the most influence (49%) on HDS use in our study. This finding is comparable with previous studies, which showed a significant impact from friends and families on HDS use [17, 18, 41]. As the internet and social media have become easy accessible nowadays, this inevitably affects the perception and knowledge of patients about disease and management [42, 43]. We found that 21.1% of the reported reasons for HDS use was information from media and the internet. We believe that the internet will become an even more extensive source of information for chronic disease patients especially DM [43]. Hence, there should be a policy for providing scientific evidence-based health information on the internet. Moreover, health care providers, including physicians, nurses, pharmacists and dietitians should update their clinical knowledge about HDS, as well as be prepared to discuss HDS effects and acknowledge adverse effects with patients.

Although the prevalence of HDS use is high, as few as 30% of patients revealed their HDS use to physicians. The primary reason for undisclosed HDS use was unawareness of this concern. Additionally, over 70% of participants exposed to HDS had no awareness of drug-herb interactions [44]. As HDS use becomes more popular, physicians should consider HDS intake to be equally as important as that of conventional medication. Our study found no statistically significant difference in any of demographic characteristic, so it may be impossible to predict the use of HDS. Thus, physicians should routinely obtain a patient’s history use of HDS, as well as screen for potential adverse effects [45].

There are a number of limitations in our study. First of all, the present study was based on patient-reported information; therefore, recall bias could occur. Secondly, this study was conducted in a specialty clinic and thus may not represent the general population, including individuals with no access to conventional medicine. Further studies should be conducted in order to estimate the impact of HDS on a national level. Continued research regarding the efficacy and safety of HDS is needed.

## Conclusions

The prevalence of HDS use in DM patients is high and can be underestimated by physicians due to undisclosed use. DM patients obtained information about HDS from their friends and family as well as the internet and other media. There should be some interventions to guide the way for evidence-based and safe HDS use. Importantly, physicians should seriously consider HDS use in DM patients and always take a complete history of HDS use.

## Additional file

Additional file 1: Herbal and dietary supplements use in DM: Questionnaires. (DOCX 507 kb)
