# Supplementary material for: Concealed use of herbal and dietary supplements among Thai patients with type 2 diabetes mellitus
Source: J Diabetes Metab Disord. 2017 Aug 23;16:36. doi: 10.1186/s40200-017-0317-3 (PMC5569552; doi:10.1186/s40200-017-0317-3)
Supplement: Additional file 1: — Herbal and dietary supplements use in DM: Questionnaires. (DOCX 507 kb) [file 40200_2017_317_MOESM1_ESM.docx]

**Herbal and dietary supplements use in DM: Questionnaires**

HN………………................. Age.................years sex ◻︎ male ◻︎ female Hometown...................

Health care scheme ◻︎ Universal coverage ◻︎ social security ◻︎ insurance

◻︎ Government/ State enterprise officer ◻︎ No Insurance (Cash)

Occupation ◻︎ government officer ◻︎ company employee ◻︎ self-employed◻︎ unemployed

◻︎ freelancer

Education ◻︎ primary school ◻︎ secondary school ◻︎ vocational certificate ◻︎ bachelor degree

◻︎ higher than bachelor degree ◻︎ N/A

Marriage status ◻︎ single ◻︎ marriage ◻︎ widow/widower ◻︎ divorce

Religion ◻︎ Buddhism ◻︎ Christian ◻︎ Muslim ◻︎ Hindu ◻︎ others....................... ◻︎ N/A

Family type ◻︎ alone ◻︎ nuclear family ◻︎ large family

Monthly income (Baht) ◻︎ < 5,000 ◻︎ 5,000 -10,000 ◻︎ 10,000 - 30,000 ◻︎ > 30,000

______________________________________________________________________________

Weight…….…… Height…………..BMI= ……………

Exercise ◻︎ none ◻︎ running ◻︎ fitness ◻︎ soccer/basketball/volleyball ◻︎ badminton/table tennis

◻︎ swimming ◻︎ yoga ◻︎ others...................... frequency (/wk) ◻︎ 0-1 ◻︎ 2-4 ◻︎ >=5 duration......year

Alcohol consumption: frequency ◻︎ never ◻︎ 0-1/mo ◻︎ 2-4/mo ◻︎ 2-3/wk ◻︎4+ /wk

Unit of alcohol ◻︎1-2 ◻︎ 3-4 ◻︎ 5-6 ◻︎ 7-9 ◻︎ 10+ Duration ……… year, stop in…………..

Smoking: ◻︎ never ◻︎ current ◻︎ former , date of stop………..Amount: ………………packs x years

______________________________________________________________________________

1. **DIABETES MELLITUS**

duration of disease…………………………SMBG ◻︎yes ◻︎no

Current medication: date___/___/___

| Metformin dose……………………………………  Sulfonylurea………………………………………..  Glinide………………………………………………  Thiazolidinedione………………………………….  Alpha-glucosidase inhibitor………………………  DPP-4 inhibitor…………………………………….  GLP-1 Analog……………………………………  SGLT 2 inhibitor………………………………… | Insulin:  Rapid acting insulin……………………………………  RI…………………………………………………………  NPH………………………………………………………  Long acting insulin……………………………………  total number of prescribing med use…………………. |
| --- | --- |

Latest Laboratory: HbA1C…………(__/__/__)FBS………(__/__/__) BUN/Cr……………(__/__/__)

UA(__/__/__): protein ◻︎negative ◻︎ micro ◻︎ positive urine protein/Cr……….

sugar ◻︎negative ◻︎positive

Complication: ◻︎ Diabetic retinopathy

◻︎ Diabetic neuropathy

◻︎ Diabetic nephropathy

◻︎ hospitalisation due to DM emergency i.e………………………………………

**2. OTHER CO-MORBIDITIES**:

◻︎ Hypertension ◻︎ CNS (stroke,………………….) ◻︎ Skeletal (OA,………………)

◻︎ Dyslipidemia ◻︎ CVS (ACS,AF,………………..) ◻︎ Cancer………………………

◻︎ CKD ◻︎ Lung (COPD,…………………) ◻︎ Other…………………………..

**3. COMPLEMENTARY AND ALTERNATIVE MEDICINE (CAM) USE**

3.1 CAM use history

- - - never use ◻︎ current use ◻︎ former use, stop for……..….mo

pattern: ◻︎ continuing user ◻︎ intermittent user

Duration……………………….mo

3.2 Number of CAM products used total……Dietary supplement…………Herbal…………

| Name of HDS | Indication | Dosage form | how do you use | dose (per day) | How often (per day/ week) | How long (month/year) | expenditure Bath/month) |
| --- | --- | --- | --- | --- | --- | --- | --- |
|  |  |  |  |  |  |  |  |
|  |  |  |  |  |  |  |  |
|  |  |  |  |  |  |  |  |
|  |  |  |  |  |  |  |  |
|  |  |  |  |  |  |  |  |
|  |  |  |  |  |  |  |  |
|  |  |  |  |  |  |  |  |
|  |  |  |  |  |  |  |  |
|  |  |  |  |  |  |  |  |

3.3 Reasons for CAM treatment (can answer more than 1)

◻︎ general health and well being

◻︎ disappointment from conventional medical therapy

◻︎ trying because of suggestion from health care providers

◻︎ trying because of suggestion from friends

◻︎ trying because of suggestion from relatives

◻︎ trying because of information from social network / social media

◻︎ they are safer than conventional medicines

◻︎ they are cheaper than conventional medicines

◻︎ treat acute conditions: pain,

◻︎ treat chronic conditions: glycemic control, BP control, insomnia, sexual problems

◻︎ other……………………………………………………………………………………….

3.4 Obtain herbal and dietary supplements from:

◻︎ buy from hospital

◻︎ buy from drug store, folk remedy shop

◻︎ buy from direct sale, magazine, newspaper, TV, internet

◻︎ provided by their family/friends

◻︎ collecting from their garden

◻︎ other …………………………………………………………………………………….

3.5 Inform the physician that you use CAM:

◻︎ Yes,

◻︎ No, because: ◻︎ they don’t ask ◻︎ no need to inform, ◻︎ other……………………

3.6 Awareness of interaction between CAM and drug: ◻︎ No ◻︎ Probably ◻︎ Yes

3.7 Effects reported by patients

◻︎ no change ◻︎ strengthening of body ◻︎ physically worse

◻︎ being in good psychological condition ◻︎ being bad

psychological condition

◻︎ feeling of relief of several symptoms

**4. Thai-version of 8-item Morisky medication adherence questionnaire**

|  | No=1 | Yes = 0 |
| --- | --- | --- |
| 1. sometimes forget to take your DM pills? |  |  |
| 2. Did not take medications in the past 2 weeks? |  |  |
| 3. Stop taking medications when feeling worse? |  |  |
| 4. Sometimes forget to bring medications when traveling? |  |  |
| 5. Did you take your DM medicine yesterday? |  |  |
| 6.Stop taking medications when well controlled? |  |  |
| 7. Feeling distressed for strictly following treatment plan? |  |  |

8. How often do you have difficulty remembering to take medications? (divide score by 4)

Never/Rarely (4), Once in a while (3), Sometimes (2), Usually (1), All the time (0)

total score = ________

Interpretation of adherence ◻︎ High (8) ◻︎ medium (6-<8) ◻︎ low (<6)
